# Post-cholecystectomy Mirizzi Syndrome

**DOI:** 10.7759/cureus.24379

**Published:** 2022-04-22

**Authors:** Amer A Alkhatib, M Ammar Kalas, Numan Balci, Abdul Manan Khaskheli, Shiva Kumar

**Affiliations:** 1 Gastroenterology and Hepatology, Cleveland Clinic Abu Dhabi, Abu Dhabi, ARE; 2 Internal Medicine, Texas Tech University Health Sciences Center El Paso, El Paso, USA; 3 Radiology, Cleveland Clinic Abu Dhabi, Abu Dhabi, ARE

**Keywords:** hepatobiliary interventions, endoscopic retrograde cholangiopancreatography (ercp), advanced gastroenterology, mirizzi syndrome, post-cholecystectomy

## Abstract

Post-cholecystectomy Mirizzi syndrome (PCMS) is characterized by symptoms of recurrent abdominal pain, jaundice, and fever in patients who underwent cholecystectomy. Imaging is crucial in the diagnosis of PCMS and Mirizzi syndrome. Imaging modalities have evolved over the years with abdominal ultrasonography, computed tomography of the abdomen, and magnetic resonance cholangiopancreatography being used in the diagnosis of PCMS and Mirizzi syndrome. The imaging findings show obstruction of the common hepatic duct due to impingement by a stone in the cystic duct or gallbladder infundibulum. PCMS management has evolved over the years with the current first-line management being endoscopic intervention. This case describes a 61-year-old male presenting with recurrent right upper quadrant pain two years after undergoing cholecystectomy due to cholelithiasis. The patient was later diagnosed with PCMS, and endoscopic management was performed with complete resolution of the symptoms.

## Introduction

Post-cholecystectomy Mirizzi syndrome (PCMS) is a clinical entity characterized by extrinsic obstruction of the common hepatic duct due to stone impaction at the level of the cystic duct or gallbladder infundibulum in patients who underwent cholecystectomy [1]. Mirizzi syndrome is a rare complication of gallstone disease, with incidence ranging from 0.05% to 2.7% in patients with cholelithiasis. It was initially described by the Argentinian surgeon, Pablo Mirizzi, in 1948 [2]. Mirizzi syndrome and PCMS are similar diseases, with the main difference between them being the history of cholecystectomy. Several cases of PCMS have been reported in the literature. Patients with PCMS are typically females and present with abdominal pain, nausea, vomiting, fever, or jaundice within a few days and up to decades after cholecystectomy [3]. This entity can be easily overlooked due to prior cholecystectomy, thus high clinical suspicion should be maintained to avoid morbidity and mortality. Herein, we present a case of a patient who suffered from biliary obstruction post-cholecystectomy and was later diagnosed with PCMS.

## Case presentation

A 61-year-old male presented to the emergency department with right upper quadrant abdominal pain of nine months duration. Past history was significant for symptomatic cholelithiasis requiring cholecystectomy two years prior to presentation. Right upper quadrant pain was non-radiating, sharp, intermittent, progressive, rated as 6/10, with no clear exacerbating or alleviating factors, and with unclear relation to oral intake. The pain was associated with nausea, jaundice, dark urine, light-colored stools, and subjective fever.

On examination, vital signs were significant for tachycardia (110 bpm) and a fever of 38.8 degrees celsius. The patient had scleral icterus without conjunctival pallor. Abdominal examination was significant for right upper quadrant tenderness without rebound tenderness or guarding.

Laboratory workup revealed: elevated alanine aminotransferase of 325 U/L (normal 17-63U/L), elevated aspartate aminotransferase of 104 U/L (normal < 40 U/L), elevated alkaline phosphatase of 165 IU/L (normal 40-129 IU/L), elevated total bilirubin of 140 micromol/L (normal range 5-21 micromol/L), elevated white blood cell count of 13,000, and a normal hemoglobin 12.8 g/L. Procalcitonin was mildly elevated at 0.94 mcg/L (normal < 0.05 mcg/L) and C-reactive protein was elevated at 58 mg/L. The panel for acute hepatitis was unremarkable.

**Figure 1:**
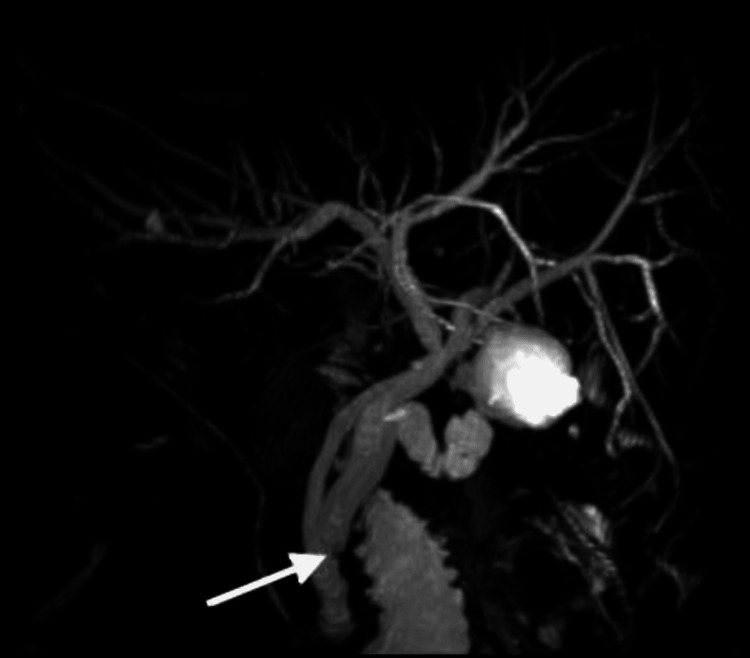
MRCP image showing low insertion of the cystic duct stump with distal calculus (white arrow) MRCP: magnetic resonance cholangiopancreatography

Compared with his prior imaging, abdominal ultrasound demonstrated an increase in the size of the convoluted cystic structure at the gallbladder bed, compatible with an increased size of gallbladder remnant, without sonographic evidence of biliary stones. To further evaluate the biliary tree, a magnetic resonance cholangiopancreatography (MRCP) was performed, which revealed a calculus in a dilated cystic duct stump with compression of the common bile duct (CBD) and upstream biliary dilatation (Figure 1).

**Figure 2:**
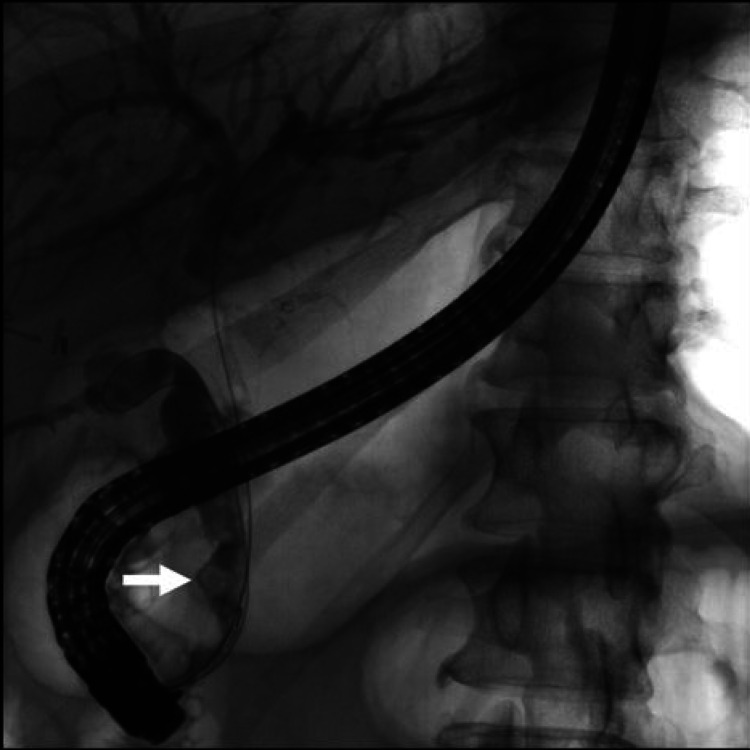
Cholangiogram showing a filling defect in a dilated cystic duct (white arrow)

The patient was started on intravenous piperacillin/tazobactam due to the suspicion of acute ascending cholangitis. Endoscopic retrograde cholangiopancreatography (ERCP) was performed and an intraprocedural cholangiogram revealed a filling defect in a distally inserted long cystic duct stump, consistent with a retained stone, resulting in partial biliary obstruction (Figure 2).

**Figure 3:**
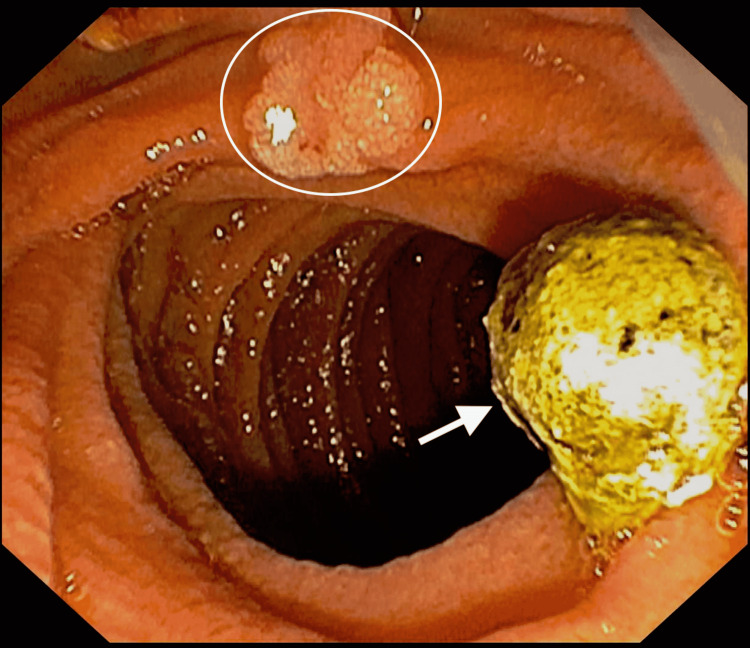
Endoscopic image of the biliary stone (white arrow) with an incidental duodenal tubular adenoma (white circle)

Following biliary sphincterotomy, the common bile duct was swept with a 9-12 mm occlusion balloon, with moderate resistance encountered at the site of cystic duct insertion. No filling defects were noted in the CBD afterward and no stones were retrieved. The cystic duct was then selectively cannulated using an Autotome™ (Boston Scientific, Marlborough, Massachusetts) and swept with the occlusion balloon, resulting in the extraction of a single large biliary stone (Figure 3).

Subsequent balloon sweeps of the CBD from the level of the bifurcation encountered no resistance. The final occlusion cholangiogram revealed no residual filling defects in the entire biliary tree, including the cystic duct stump. Excellent drainage of bile and contrast was observed. The overall cholangiographic findings were consistent with PCMS. The patient’s symptoms resolved promptly thereafter, with no subsequent recurrence.

The patient was seen in the outpatient clinic after six weeks with no recurrence of his symptoms. Liver biochemical tests revealed normalization of aspartate aminotransferase (20 U/L), alanine aminotransferase (29 U/L), and alkaline phosphatase (78 IU/L). Indirect hyperbilirubinemia was present. Therefore, a workup for hemolysis was pursued after which he was diagnosed with glucose-6-phosphate dehydrogenase (G6PD) deficiency. He was referred to the hematology clinic for further management.

## Discussion

PCMS and Mirizzi syndrome are complications of cholelithiasis that ensue when a gallbladder stone gets lodged in Hartmann's pouch or the cystic duct, leading to extrinsic compression of the biliary system. Mirizzi syndrome can be complicated by a cholecystobiliary or cholecystoenteric fistula, gallbladder empyema, and gallbladder perforation with subsequent sepsis [2]. Classification of Mirizzi syndrome is dependent upon the presence or absence of a cholecystobiliary fistula and its extent. Type I is characterized by compression of the distal common hepatic duct with an impacted stone at the level of the gallbladder neck or infundibulum without fistula formation. Type II is characterized by fistula formation involving less than a third of the common bile duct circumference. Type III also has fistula formation but involves one-third to two-thirds of the common bile duct circumference. Type IV is characterized by obliteration of the entire common bile duct wall [4]. A classification system for PCMS does not exist, however, experts utilize the Mirizzi syndrome classification system due to the similarities of the conditions' pathophysiology.

PCMS has been reported in more than 65 cases in the literature [3]. It is estimated that approximately 10%-15% of patients who underwent cholecystectomy suffer from recurrent or persistent biliary manifestations and can even develop new symptoms such as diarrhea. The aforementioned symptoms can occur individually or together and are considered part of the post-cholecystectomy syndrome. The post-cholecystectomy syndrome is a result of a variety of functional or structural defects, which can be intrinsic or extrinsic to the biliary tract, one of which is PCMS [5]. The estimated incidence of PCMS is <2.5% over a two-year interval (post-cholecystectomy) and up to 7% over a four-year interval [6-7]. PCMS is more likely to occur in females (approximately 60%) with an average age at diagnosis of 49 years. Risk factors for PCMS include laparoscopic subtotal cholecystectomy, low insertion of the cystic duct, and long cystic duct remnant (> 1 cm) [8].

Typically, patients with PCMS present with right upper quadrant pain, jaundice, nausea, and vomiting. Fever can occur in patients with concomitant acute cholangitis [3].

PCMS and Mirizzi syndrome are similar diseases, with the primary difference being the history of cholecystectomy. Therefore, clinical presentation, diagnosis, and lab values are similar between the two entities. Clinically, the primary difference between the two entities is that Mirizzi syndrome can present with acute cholecystitis (approximately one-third of patients), which does not occur in PCMS [9].

Laboratory evaluation of PCMS reveals elevations in alkaline phosphatase (ALP), gamma-glutamyl transferase, and direct hyperbilirubinemia in the majority of patients. In a review of 25 cases of PCMS, approximately 45% had elevated aspartate aminotransferase (AST) and alanine aminotransferase (ALT) with non-specific patterns. Of those, approximately 55% had moderate elevations in AST and ALT while the remainder had severe elevations in AST and ALT (>10x upper limit of normal). Leukocytosis can occur in patients with accompanying acute cholangitis [3].

The diagnosis requires the presence of imaging findings and can be done through abdominal ultrasonography, CT of the abdomen with contrast, or MRCP. Ultrasonographic findings include intrahepatic biliary dilation, impacted stone at the gallbladder neck, and sudden normalization of the common bile duct below the stone level [10]. Abdominal CT can aid with the identification of possible underlying malignancy, however, it does not yield any additional diagnostic benefit when compared to abdominal ultrasonography [11]. MRCP is a modality that has high sensitivity and can aid in the diagnosis, as it can assess the extent of pericholecystic inflammation and can help exclude other possible etiologies such as underlying malignancy. Moreover, T2-weighted MRCP can also differentiate inflammatory masses from neoplastic ones and hence can have more diagnostic value when compared to abdominal ultrasonography or abdominal CT [8,12]. Nevertheless, ERCP is often needed for the confirmation of the diagnosis and for the evaluation of a possible cholecystobiliary fistula. In cases where ERCP is not possible or failed, percutaneous transhepatic cholangiography can be performed [10].

Physicians dealing with cases suspicious for Mirizzi syndrome should consider gallbladder cancer and cholangiocarcinoma in the differential diagnosis. A retrospective study was done by Redaelli CA et al., which evaluated the incidence of gallbladder carcinoma in patients with Mirizzi syndrome. The study found that 27.8% of patients with Mirizzi syndrome were diagnosed incidentally with gallbladder carcinoma. The study also evaluated the incidence of gallbladder carcinoma in patients with cholelithiasis, which was found to be approximately 2%. The difference between the incidence of gallbladder carcinoma in patients with Mirizzi syndrome and patients with cholelithiasis but without Mirizzi syndrome was found to be statistically significant (p-value < 0.001) [13]. Moreover, a retrospective study done by Clemente G et al. assessed the incidence of gallbladder malignancy in patients diagnosed with Mirizzi syndrome. The study reported that approximately 33% of patients diagnosed with Mirizzi syndrome had underlying gallbladder malignancy [14]. One of the main limitations of both studies was the small sample size because of the low incidence and prevalence of Mirizzi syndrome. Due to the rarity of PCMS, no studies have evaluated the incidence of malignancy in patients with PCMS.

The standard treatment for Mirizzi syndrome is surgical intervention, namely, cholecystectomy. ERCP can be utilized as a temporizing measure in patients with cholangitis while awaiting definitive surgical intervention [2]. As patients with PCMS already underwent cholecystectomy, the mainstay of treatment is through endoscopic intervention.

Treatment of PCMS has evolved from surgical intervention to ERCP, where selective cannulation of the biliary duct is followed by directing the wire/biliary catheter to the cystic duct. Once access to the cystic duct is achieved, extraction of the stone is carried out using a balloon, basket, mechanical lithotripsy, or electrohydraulic lithotripsy [3]. In cases where cystic duct access or attempts to clear the obstruction fails, a temporary stent can be placed, with its proximal end in the proximal common hepatic duct and its distal end in the duodenum, which will aid in decompressing the biliary tree. This management strategy is temporary, as plastic stents often occlude or migrate within weeks to months from placement [15]. In cases where the trans-papillary biliary approach fails, access can be achieved through the proximal biliary tree with the utilization of endoscopic ultrasound. A guidewire can then be passed, after which a self-expanding covered metallic stent is placed to relieve the biliary obstruction. Moreover, percutaneous biliary decompression, surgery, and laparoscopic ERCP can be of use in cases unamenable to endoscopic interventions [3].

## Conclusions

PCMS is a rare entity that can be easily overlooked. Therefore, medical providers should have a low threshold to consider the diagnosis in patients presenting with abdominal pain and jaundice post cholecystectomy. In our case, the patient presented with symptoms of biliary obstruction two years following his cholecystectomy. Further imaging and endoscopic workup revealed evidence of PCMS. Clinical awareness about the condition, coupled with appropriate imaging studies, helps in confirming the diagnosis. Abdominal ultrasonography and MRCP were utilized successfully in our report. Prompt treatment can prevent severe complications such as cholecystodochal fistula formation, acute cholangitis, and severe sepsis. Our patient underwent endoscopic intervention with complete symptom resolution. Endoscopic management of PCMS is feasible and is preferred over surgical treatment.
